# Improving sodium laser guide star brightness by polarization switching

**DOI:** 10.1038/srep19859

**Published:** 2016-01-22

**Authors:** Tingwei Fan, Tianhua Zhou, Yan Feng

**Affiliations:** 1 Shanghai Institute of Optics and Fine Mechanics, Chinese Academy of Sciences, and Shanghai Key Laboratory of Solid State Laser and Application, Shanghai 201800, China; 2 University of the Chinese Academy of Sciences, Beijing 100049, China

## Abstract

Optical pumping with circularly polarized light has been used to enhance the brightness of sodium laser guide star. But the benefit is reduced substantially due to the precession of sodium atoms in geomagnetic field. Switching the laser between left and right circular polarization at the Larmor frequency is proposed to improve the return. With ESO’s laser guide star system at Paranal as example, numerical simulation shows that the return flux is increased when the angle between geomagnetic field and laser beam is larger than 60°, as much as 50% at 90°. The proposal is significant since most astronomical observation is at angle between 60° and 90° and it only requires a minor addition to the delivery optics of present laser system.

What is the nature of the dark matter and dark energy and how many Earth-like and habitable planets populate the Galaxy are the examples of big questions to be answered in astronomy in next decades^1^. As a powerful tool for comprehensive understanding of the Universe, the ground-based large-aperture telescopes advanced rapidly with higher sensitivity and higher spatial resolution^2^ and achieved significant observations^3^. The high spatial resolution is possible thanks to a technology known as adaptive optics (AO), which corrects blurred images introduced by atmospheric turbulence in real time^4^. Laser guide star (LGS) technique enables the creation of artificial beacons, so that AO observations of astronomical targets that are themselves too faint to be used for such measurement can be performed^5^. The so-called sodium LGS is generated by exciting the mesospheric sodium atom layer at 90 km above the sea level with a 589 nm laser, which is widely used at present astronomical telescopes and considered best choice of beacons^6^,^7^.

The brightness of the artificial star is one of the key parameters determining the performance of AO. Improving sodium excitation efficiency by optimizing the spectral, temporal and polarization characteristics of the 589 nm laser beam has been a focus of study in the astronomical community. The use of circularly polarized laser was proposed to have brighter guide star utilizing the optical pumping process. The increase in return photons for circularly over linearly polarized light has been reported both experimentally and theoretically^8^,^9^,^10^.

The benefits of optical pumping are substantially reduced with the presence of geomagnetic field, because the sodium atoms precess along the magnetic field^11^,^12^,^13^. The precessing atoms are reoriented faster than the time it takes to establish a beneficial distribution. Precession of the angular momentum does not occur when the laser beam is parallel to the magnetic field, but is maximized for the perpendicular case. Unfortunately, large angles are more typical in astronomical observing. Thomas J. Kane *et al*. proposed to use a laser source pulsed at the Larmor frequency for improving the brightness of sodium LGS^14^. It works because it allows the laser light to interact with the atoms at a fixed point in the precession cycle. Their simulation shows a photon return double that of an optimized cw laser at the same average power for large angles between laser beam and geomagnetic field. But sodium guide star lasers pulsed at this frequency range are not available and technically challenging to develop.

In this paper, we propose a method of “magnetically resonant pumping” with currently available cw laser to overcome the problem induced by the magnetic field. Magnetically resonant pumping is achieved by switching the polarization of the cw guide star laser between left and right circular polarization at a rate of Larmor frequency. Numerical simulation with ESO’s laser guide star system at Paranal as example shows that the return flux can be increased at most astronomical observation angle and as much as 50%. Note that the gain in LGS brightness is achieved with only minor modification to the optics after the currently installed cw laser.

## Results

### The proposal

**Figure 1:**
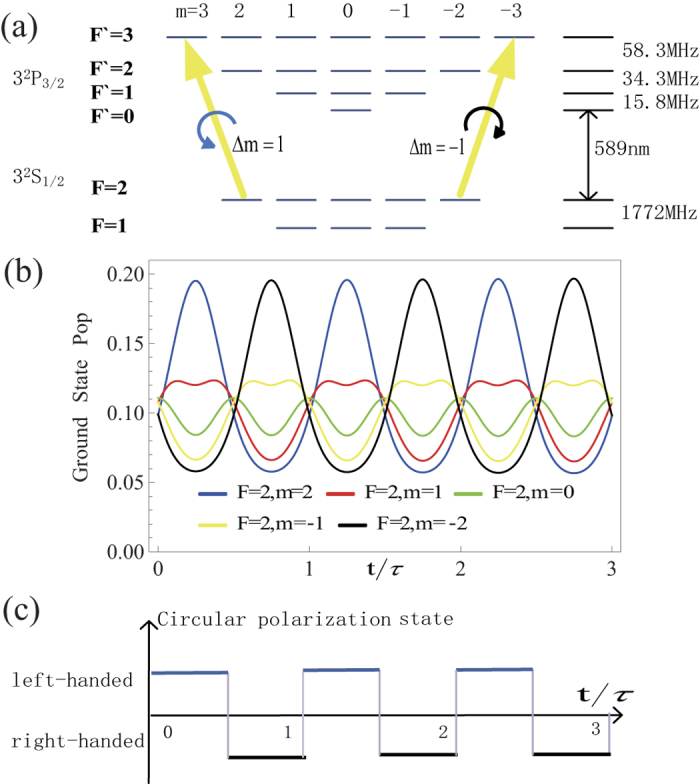
Schematic diagram of the polarization switching method for improving the sodium excitation. (**a**) the sodium D_2_ transitions and energy levels. (**b**) Population dynamics on the five F = 2 ground state magnetic sublevels when the angle between laser beam and magnetic field *θ* is *π*/2. *τ* is the period of Larmor precession. (**c**) The timing diagram of laser polarization switching.

The proposal of magnetically resonant pumping by polarization switching is sketched in Fig. 1. Figure 1a illustrates the energy levels of 3^2^ P_3/2_ and 3^2^ S_1/2_ involved in the sodium D_2_ transitions^15^. The D_2_a transition from F = 2 ground state is usually chosen in LGS application for higher oscillation strength. When the laser beam is left circular polarization (σ+), population migrates toward the 3^2^ S_1/2_ (F = 2, m = 2) substate due to transition selection rules. Finally the atoms cycle on the transition from 3^2^ S_1/2_(F = 2, m = 2) to the 3^2^ P_3/2_(F = 3, m = 3), which has the highest transition cross section, and effectively become a two-level system^16^. This beneficial redistribution of angular momentum states is called “optical pumping”.

However, sodium atoms with angular momentum precess around the geomagnetic field. The population oscillates between m substates at a Larmor frequency. Therefore, the benefits of optical pumping are reduced to a large extent, especially for the case of large angle between the beam and the magnetic field (*θ*). The benefit of optical pumping is almost completely eliminated if the beam is perpendicular to the field. Unfortunately, angles closer to perpendicular are more representative in astronomical observing.

We propose a method to overcome the influence of geomagnetic field by switching the polarization of a cw guide star laser between left and right circular polarization at a rate of Larmor frequency. Figure 1c illustrates the proposed polarization switching pattern, the Larmor precession period in a magnetic field B equals *τ* = (2*πћ*)/(*g*_F_*μ*_B_B), where μ_B_ is the Bohr magneton (9.274 × 10^−24^ J/T), and g_F_ is the Landé factor. The polarization of the laser switches between left and right circular at a half of Larmor period. Figure 1b shows the corresponding population dynamics on the magnetic sublevels when *θ* = π/2. The groundstate populations oscillate at the same period of Larmor precession. When the 3^2^ S_1/2_(F = 2, m = 2) substate has higher than average population, the laser polarization is *σ*+. The laser excites sodium atom from 3^2^ S_1/2_(F = 2, m = 2) to 3^2^ P_3/2_(F = 3, m = 3), which has the highest transition strength. When the 3^2^ S_1/2_(F = 2, m = −2) substate has higher population, the laser polarization is right circular polarization (*σ*−). The laser then excites sodium atom from 3^2^ S_1/2_(F = 2, m = −2) to 3^2^ P_3/2_(F = 3, m = −3), which again has the highest transition strength. By this way, the sodium atoms are excited at a magneto-optical resonance. Higher excitation rate and fluorescence return is expected.

Such polarization switching can be implemented in existing cw laser guide star systems rather readily. A quarter wave plate is usually inserted in the laser delivery path to generate a circular polarized beam from a linearly polarized beam for optical pumping. To achieve polarization switching, one can exchange the quarter wave plate with an electro-optic phase modulator, and align the polarization of the laser at 45° angle with the optical axis of the electro-optic crystal. Applying voltage on the crystal will produce a variable phase delay between the ordinary and extraordinary field components. Therefore, if one applies appropriate voltage on the crystal to produce a phase delay of −π/2 and π/2 alternately, the laser will be switched between left and right circular polarization. The required switching frequency is on the level of 100 kHz. Such electro-optic phase modulator is commercially available.

### The numerical model

Simulations of the interaction of sodium atoms with laser have been implemented by many researchers^10^,^17^,^18^,^19^. To calculate the observed fluorescence, the evolution of the atoms is modeled using the optical Bloch equations for atomic density matrix, which describes the statistical state of the atomic ensemble. The simulation is based on the open-source LGSBloch package for Mathematica^20^. Program for the polarization switching is implemented to carry out the calculation. A piecewise function is used to define the polarization switching as instantaneous square-wave switching. Similar results are expected for square wave switching with nanosecond leading and falling edges. Sine-wave modulation of polarization is also simulated, but the improvement in the return flux is not obvious. So sine-wave polarization modulation is not to be discussed here. The fluorescent photon flux per solid angle emitted at a given direction is found as the expectation value of a fluorescence operator.

In our simulation, part of atomic, atmospheric, and mesospheric parameters are standard parameters as defined in Table 1 of ref. 10. Taking ESO’s laser guide star system at Paranal as an example, the laser parameters are as follow: laser power centered at D_2_a line is 18 W, the repumping light is 2 W (power fraction q = 10%), and the laser linewidth is <5 MHz. The geomagnetic field at 90 km above Paranal is 0.22750 G, which means the precession frequency of sodium atoms is 159.207 kHz. Table 1 lists all simulation parameters relevant to the simulation in this paper. Furthermore, the return flux in our simulation results is defined as *ψ* = *Ψ*/I in unit of photons/s/sr/atom/(W/m^2^), which is the number of photons per unit time, per solid angle, per atom and per laser irradiance spontaneously emitted in the direction of the launch telescope, as observed in the mesosphere.

### Simulation results and discussion

**Figure 2:**
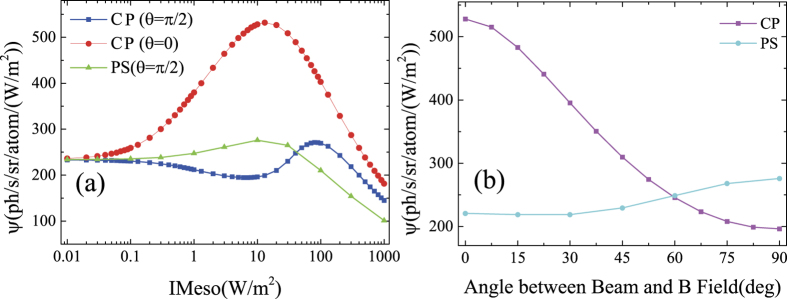
Calculated return flux for different conditions. (**a**) Specific return flux *ψ*(I) for different polarization conditions and different angles between laser beam and magnetic field *θ*. CP stands for circular polarization, and PS stands for polarization switching. (**b**) The calculated return flux *ψ* versus *θ* at a laser power intensity of 10 W/m^2^ with repumping power fraction of 0.1.

The return flux versus laser irradiance is calculated for different polarization states and different angles between laser beam and geomagnetic field. Some of the results are shown in Fig. 2. At a low irradiance level below 0.01 W/m^2^, the distribution of sodium states is not affected by the laser. The best return is achieved with a laser exactly centered on the peak of the sodium D_2_a line at 589.159 nm. The goal of optimization is to maintain or even exceed the return flux at low irradiance level over a wide range of laser irradiance. In this case, the return signal can increase linearly or even faster with increasing laser power.

When the laser is circularly polarized and projects parallel to the geomagnetic field (*θ* = 0), Larmor precession is absent and the return flux increases with laser power and reaches a maxima at around 10 W/m^2^ due to optical pumping. However, when the laser is perpendicular to the geomagnetic field (*θ* = *π*/2), the effect of optical pumping is completely suppressed by Larmor precession. The return flux decreases with respect to laser power and reaches a minima at 10 W/m^2^ instead. Also shown in Fig. 2(a) is the results for the proposed polarization switching case at *θ* = *π*/2. The return flux increases with respect to laser power and maximizes at 10 W/m^2^. The result shows that polarization switching can indeed compensate for the inhibition on optical pumping by Larmor precession. Figure 2(b) shows the return flux versus angle between laser beam and geomagnetic field *θ* for the cases of cw circularly polarization and polarization switching. When *θ* is small, circular polarization is better than polarization switching. When *θ* is greater than 60°, the situation reverses. The gain in the return flux with polarization switching increases with angle *θ*, and is as much as 50 % at an angle of 90°.

Although the range of angle in which polarization switching has gain is only from 60° to 90°, most telescopes operate in the 60°–90° range in most of the time. Therefore, the proposal is significant, considering it only requires a minor modification to the present cw guide star laser systems installed at telescopes like VLT at Paranal, Chile. In case that the telescope operates below 60° and the influence of Larmor precession is small, one can simply turn off the polarization switching and gain the benefit of optical pumping.

**Figure 3:**
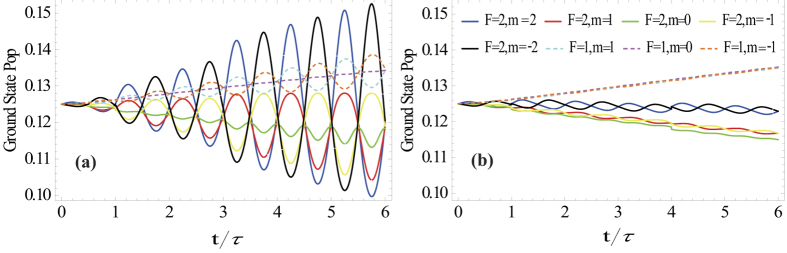
Occupation probabilities of the eight ground state’s magnetic sublevels as functions of the normalized time 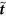 = t/τ for *θ* = *π*/2 and *I* = 10 W/m^2^. **(a)** Polarization switching at Larmor frequency of 159.207 kHz, (**b**) cw laser of single polarization state.

The population dynamics is investigated to understand the mechanism of return flux improvement better. Figure 3(a,b) shows occupation probabilities evolution of the eight ground state magnetic sublevels for *θ* = *π*/2 after switching on the laser, with and without polarization switching, respectively. Without polarization switching, populations on the five F = 2 magnetic sublevels decrease gradually, while populations on the three magnetic sublevels of F = 1 increase. Population oscillation is seen for the F = 2 sublevels, which is the sign of Larmor precession. The population becomes stable in about 50 Larmor periods. With polarization switching, the populations of the magnetic sublevels (F = 2, m = 2) and (F = 2, m = −2) oscillate and become the highest alternatively during one Larmor period. The laser switches between left and right circular polarization within one Larmor period correspondingly. That is why it can excite the sodium atom more efficiently. The populations on the F = 2 and m = 1, 0, −1 sublevels also oscillate but in a smaller scale, and decline slowly. Populations on the three magnetic sublevels of F = 1 increase gradually as well, which indicates that the down pumping effect is obvious when the repumping power fraction is 0.1. The repumping power is not high enough.

**Figure 4:**
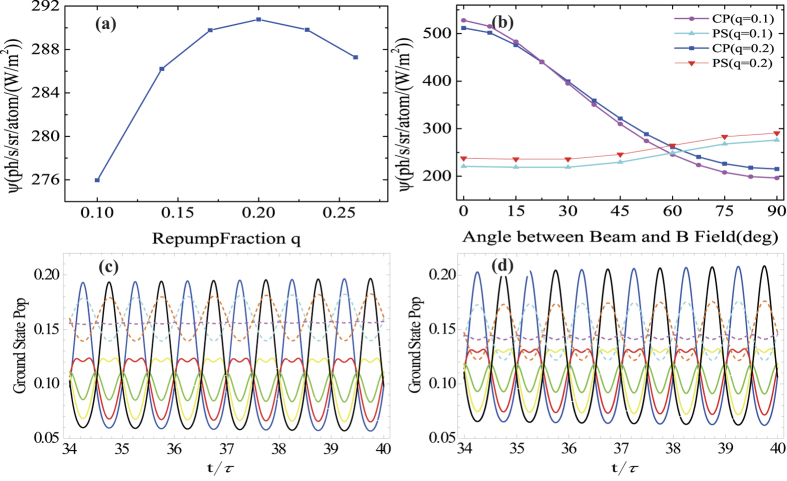
Specific return flux in case of *θ* = π/2 and *I* = 10 W/m^2^. (**a**) return flux *ψ* as function of repumping fraction *q*, and (**b**) calculated return flux *ψ* versus angle between laser beam and magnetic field for both q = 0.1 and q = 0.2. Occupation probabilities evolution of the eight ground state’s magnetic sublevels at (**c**) q = 0.1 and (**d**) q = 0.2. The figure (**c**,**d**) share the same legend as in Fig. 3(b).

Keeping the total laser power constant at 20 W, the return flux versus repumping fraction *q* is then calculated and shown in Fig. 4(a). The optimum repumping laser fraction is found to be about 20%. Since photon return does not vary significantly with repumping ratio q, only about 5% for q value from 0.1 to 0.2, in practice q can be optimized empirically. The return flux versus angle (as Fig. 2(b)) is calculated again for the case of 20% repumping. The results are shown in Fig. 4(b). For both cw circularly polarization and polarization switching cases, the return fluxes increase slightly at large *θ*. The benefit of the polarization switching remains at angle >60°. Fig. 4(c,d) shows the population dynamics of the eight ground state’s magnetic sublevels in the cases of q = 0.1 and q = 0.2 for comparison. It is clear that, for the q = 0.2, populations on the three magnetic sublevels of F = 1 are relatively low. The populations on the F = 2 sublevels (m = 2 and −2 magnetic sublevels in particular) are higher due to better repumping, which explains the higher return flux.

**Figure 5:**
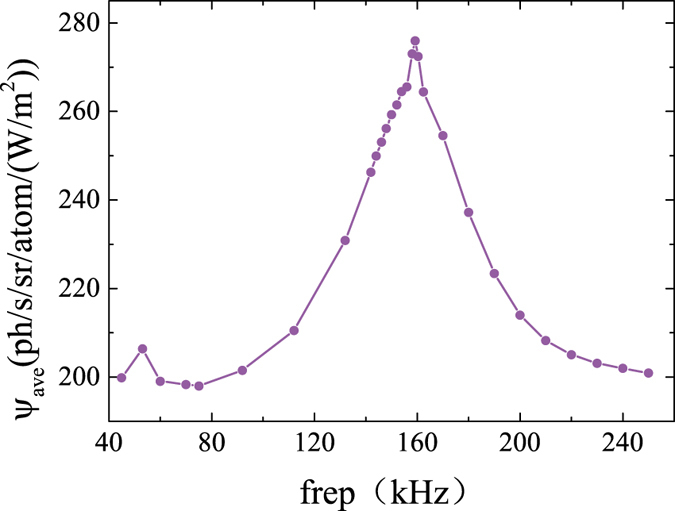
ψ as a function of the polarization switching frequency at I = 10 W/m^2^ and *θ* = *π*/2.

In above calculations, the polarization switching frequency is set to resonant with the Larmor precession. However, the Larmor frequency is proportional to the local geomagnetic field at the sodium layer, and therefore varies with the laser projection direction. Is it necessary to adjust the switching frequency with different observing direction? The dependence of return flux on the polarization switching frequency is therefore calculated, the result is shown in Fig. 5.

The return flux peaks at Larmor frequency *ƒ*_*L*_ as expected. Interestingly, there is a small peak at *ƒ*_*L*_*/*3 as well, but no peak at *ƒ*_*L*_*/*2. This observation is different from the pulsed laser pumping method, where the return flux peaks at all the subharmonics of Larmor frequency^14^. When the frequency is *ƒ*_*L*_, the laser switches the polarization every half Larmor period as illustrated in Fig. 1. The population dynamics is driven synchronously. If the frequency is *ƒ*_*L*_*/*2, the laser switches every full Larmor period. As seen in Fig. 1, the groundstate populations oscillate a full circle in one Larmor period, so the enhancement in sodium excitation is lost. If the frequency is *ƒ*_*L*_*/*3, the laser switches the polarization every 1.5 Larmor period. A reduced resonant peak is expected. The full width half maximum width of D_2_a line “polarization magneto-optical resonance” is found to be about 40 kHz. In Paranal, a ± 30° scanning range of telescope means a geomagnetic field variation of about 100 nT at sodium layer^21^, which corresponds to a Larmor frequency change of only 0.7 kHz. The wide polarization magneto-optical resonance is beneficial for the Na laser guide star application, because it suggests that there is no need to adjust the switching frequency when observing astronomical objects at different directions.

The magneto-optical resonance of Na excitation has been proposed for remote measurement of magnetic field. In ref. 22, J. M. Higbie *et al*. considered to measure the magnetic field at the height of sodium layer by shining a pulsed 589 nm laser and resolving the resonant pulse repetition rate. Since Na guide star laser pulsed at Larmor frequency (a few hundred of kHz) is not available at present, chopping of a cw laser is to be used as excitation source. Since the optimum laser duty cycle is about 20%, according to their calculation, a majority (80%) of the laser power is lost. Obviously, polarization switching of currently available cw laser can also be used to measure the magnetic field by finding the Larmor frequency. It has an advantage of no laser power lose and, therefore, higher return signal, which is technically important for such photon hungry application.

In conclusion, we propose to improve the brightness of the cw sodium LGS by switching the laser between left and right circular polarization at a Larmor frequency. For the cw laser system installed at ESO Very Large Telescope at Paranal as example, numerical simulation shows that the return flux is improved when the angle between geomagnetic field and laser beam is larger than 60°, by 50% at an angle of 90°. Polarization switching can partly compensate the inhibitory effect of Larmor precession on optical pumping, and brighter sodium laser guide star can be generated. Since most astronomical observation is at angle between 60° and 90° and it only requires a minor addition to the laser launch optics, the polarization switching method is significant for astronomical community and telescope sites where cw Na guide star has been or will be installed. A by-product of the proposal is that the polarization switching method may also be a preferred method for remote measurement of magnetic field.

## Additional Information

**How to cite this article**: Fan, T. *et al*. Improving sodium laser guide star brightness by polarization switching. *Sci. Rep.* **6**, 19859; doi: 10.1038/srep19859 (2016).

## Figures and Tables

**Table 1:** Simulation parameters and their standard nominal values.

Variable name (Symbol)	Standard value
Atomic, atmospheric and mesospheric parameters	
Geomagnetic field in mesosphere (B)	0.22750 G
Average mesospheric temperature (T_Na_)	185 K
Na beam dwell velocity (v_γ_)	38 m/s
Beam atom exchange rate (γ_ex_)	1/(6.0 ms)
Na–N_2_ v.c.c.^†^ cross section (σ_Na−N2_)	0.72 × 0^−14^ cm^2^
Na–O_2_ v.c.c.^†^ cross section (σ_Na−O2_)	0.70 × 10^−14^ cm^2^
Weighted v.c.c.^†^ rate (γ_vcc_)	1/(35 μs)
Na–O_2_ spin exchange cross sect. at T_Na_ (σ^S^_Na−O2_)	0.50 × 10^−14^ cm^2^
Weighted spin-exchange rate at T_Na_ (γ_S_)	1/(245 μs)
Recoil frequency Shift of sodium atoms (Ω_NaRecoil_)	50 kHz
**Laser parameters**	
Launched laser power in air (P)	20 W
Central D_2_ vacuum wavelength (λ)	589.159 nm
Laser FWHM linewidth (Δf)	<5 MHz
Repumping power fraction (q)	0.1
Repumping frequency offset (Δf_ab_)	1.7178 GHz
